# Lignin Degradation Potential of Novel *Streptomyces* Isolates from the *Juniperus turcomanica* Rhizosphere via Broad-Spectrum Enzymatic Activities

**DOI:** 10.3390/microorganisms14102347

**Published:** 2026-10-14

**Authors:** Keremli Saparmyradov, Abdulla A. Yergeshov, Bakhtiyar R. Islamov, Altyn K. Rahmanova, Durdymyrat G. Gadamov, Natalia V. Trachtmann

**Affiliations:** 1 Federal Research Center, Kazan Scientific Center of Russian Academy of Science, Kazan 420111, Russia; abdulla.ergeshov@mail.ru (A.A.Y.); b.islamov@knc.ru (B.R.I.); n.trahtman@knc.ru (N.V.T.); 2 International Scientific—Technological Park of the Academy of Sciences of Turkmenistan, Ashgabat 744032, Turkmenistan; rahmanovaaltyn55@gmail.com (A.K.R.); gadamov2022@gmail.com (D.G.G.)

**Keywords:** *Streptomyces*, hydrolytic enzymes, 16S rRNA, antagonism, ligninolytic activity

## Abstract

This study provides a comprehensive characterization of *Streptomyces* isolates associated with the rhizosphere of the centenarian *Juniperus turcomanica*. Screening for ligninolytic activity revealed that isolates—ATM9, ATM14, ATM15, ATM16, ATM18, and ATM19 synthesize enzymes capable of decolorizing methylene blue and modifying lignosulfonates. Isolates ATM16 and ATM18 demonstrated the highest efficiency in lignosulfonate decomposition, with lignosulfonate becoming undetectable in their culture broth. In the straw lignin degradation process, isolates ATM15 and ATM16 showed the most significant reductions in lignin content (44.2% and 54.8%, respectively), demonstrating promising biodegradation potential. Majority of isolates demonstrated antagonistic activity against fungal strains, while ATM19 completely inhibited the growth of all phytopathogenic fungi tested. Taxonomic identification based on 16S rRNA gene sequencing confirmed that all isolates belong to the genus *Streptomyces*, with species including *S. shaanxiensis* (ATM9), *S. chryseus* (ATM14), *S. narbonensis* (ATM15), *S. venezuelae* (ATM16), *S. spiroverticillatus* (ATM18), *S. longhuiensis* (ATM19), and *Streptomyces* sp. (ATM24). Considering our data, *S. venezuelae* ATM16 and *S. spiroverticillatus* ATM18 stand out as the most promising strains for biotechnological applications due to their ligninolytic and antagonistic activity against fungal phytopathogens. Furthermore, this study provides the first report on enzymatic and antifungal activities for the recently proposed species *S. longhuiensis* (ATM19) and *S. shaanxiensis* (ATM9).

## 1. Introduction

The genus *Streptomyces* consists of obligate aerobic gram-positive spore-forming actinobacteria, with filamentous growth [1]. These bacteria can be found in Antarctic soils [2], deserts [3], and aquatic ecosystems, including deep-sea environments [4], due to their ability to adapt to extreme conditions. The primary ecological role of most *Streptomyces* species is the decomposition of complex organic matter and the recycling of nutrients in soil, water sediments and decomposing wood [5]. The widespread distribution of *Streptomyces* in soil is not surprising, as the “earthy” smell of moist soil is actually caused by geosmin, a substance produced by *Streptomyces* species [6]. *Streptomyces* sp. strains have large genomes, with a high GC content often exceeding 70% and contain a linear mini-chromosome and plasmids. *Streptomyces* strains harbor numerous biosynthetic genes, coding for a wide variety of secondary metabolites that could be useful in medicine, veterinary and agriculture [7,8,9].

Agricultural biopreparations of streptomycetes enhance plant growth by facilitating nutrient acquisition, particularly by solubilization and mobilization of phosphates from soil phytate complexes [10], as well as directly stimulating plant development by synthesizing phytohormones, including auxin, gibberellin, and abscisic acid [11]. Many *Streptomyces* strains act as antagonists against plant pathogens such as *Fusarium oxysporum*, *Fusarium solani*, *Rhizoctonia solani*, *Botrytis cinerea*, *Alternaria solani*, *Alternaria alternata*, and *Colletotrichum gloeosporioides* [12,13].

*Streptomyces* also attract significant interest due to their ability to produce a broad spectrum of hydrolytic enzymes, such as amylases, cellulases, laccases, lipases, lignin peroxidases and proteases [14]. Ligninolytic enzymes of streptomycetes could significantly aid in the degradation of lignin, which is one of the most abundant and at the same time recalcitrant plant biopolymers, and its derivatives [15]. Conventional physical or physico-chemical methods of lignin degradation often require harsh conditions and are associated with high environmental risks. Alternatively, microorganisms can perform lignin decomposition/modifications under mild, environmentally friendly conditions. In particular, several strains of *Streptomyces* have a wide-range enzymatic arsenal to degrade 10–89% of lignin by laccase (Lac), manganese peroxidase (MnP), dye-decolorizing peroxidase (DyP), and lignin peroxidase (LiP) activities that reduce molecular weight of this plant polymer. Therefore, the discovery of new *Streptomyces* strains offers a promising way to turn lignin into valuable aromatic compounds, such as vanillin, catechol, and muconic acid for pharmacy polymer industries [16].

Rhizosphere of healthy long-lived plants may contain unique strains with high potential for both lignin decomposition and plant growth promotion. Moreover, harsh conditions under which the plants grow might enrich the rhizobiome with microorganisms adapted to extreme conditions. *Juniperus turcomanica* from the arid region near the Makhtymkala village in Turkmenistan, characterized by low humidity and high air temperatures, is a well-known compound for folk medicine [17]. As a centenarian plant, with some specimens reaching an estimated age of 1000 years, it is thought that rhizosphere of *J. turcomanica* harbors a stable and adapted microbiome. We hypothesized that the root microflora associated with centenarian *J. turcomanica* may be enriched in *actinobacteria* capable of decomposing the lignin-rich dead outer layers of healthy living plants while thriving under these extreme conditions without harming the host plant.

In this work, we have isolated *Streptomyces* strains associated with 250–300-year-old *J. turcomanica* roots, and analyzed their ability to inhibit fungal phytopathogenic strains and their capability to produce lytic enzymes, with a focus on ligninolytic activity.

## 2. Materials and Methods

### 2.1. Solutions, Media and Strains Used in This Study

Isolation of *Streptomyces* strains was carried out on Gause’s No. 1 agar medium (starch, 20 g/L; K_2_HPO_4_, 0.5 g/L; MgSO_4_·7H_2_O, 0.5 g/L; KNO_3_, 1 g/L; NaCl, 0.5 g/L; FeSO_4_·7H_2_O, 0.005 g/L; agar, 20 g/L; pH 7.2–7.4). Nystatin (Biosintez, Moscow, Russia) at 500,000 U per L was added to Gause’s No. 1 agar to prevent growth of fungi. For suspending samples or bacteria/fungal cultures, phosphate-buffered saline (PBS) (Servicebio, Wuhan, China) was used. Mineral medium (MMA) containing K_2_HPO_4_ 5.8 g, KH_2_PO_4_ 3 g, (NH_4_)_2_SO_4_ 1 g, glycerol 10 g and agar 15 g, all per 1 L, was used to test cellulolytic and proteolytic activities, for this MMA was amended with 1% carboxymethyl cellulose (CMC) (CDH, New Delhi, Delhi, India) and 1% of sterilized skim milk powder (10%) solution (CDH, New Delhi, Delhi, India). Lignin degradation was tested in liquid mineral medium (MMB). For amylase activity testing, Gause’s No. 1 agar medium was used. To assess lipase activity medium containing peptone, 10 g/L; NaCl, 5 g/L; CaCl_2_·2H_2_O, 0.1 g/L; Tween 80, 10 mL/L; agar, 15 g/L was used [18]. Phytase activity was evaluated on phytate screening agar (glucose, 20 g/L; CaCl_2_, 2 g/L; NH_4_NO_3_, 5 g/L; MgSO_4_·7H_2_O, 0.5 g/L; KCl, 0.5 g/L; MnSO_4_·7H_2_O, 0.01 g/L; FeSO_4_·7H_2_O, 0.01 g/L; sodium phytate, 5 g/L; agar, 15 g/L) [18]. Methylene blue (DIA-M, Moscow, Russia) decolorization test was carried out in Gause’s No. 1 and King B (Condalab, Madrid, Spain) broths. To evaluate ligninolytic activity, a liquid medium containing lignosulfonate 5 g/L; NaCl 0.5 g/L; MgSO_4_·7H_2_O 0.5 g/L was prepared. All reagents used for media preparation were purchased from Reachim (Moscow, Russia) if not otherwise stated. All strains used in this study are present in the Table 1.

**Table 1:** Strains used in this study.

Strain	Description	Source, Reference
*Alternaria alternata* F-3047	Isolated from potato (*Solanum tuberosum*)	ARCM *
*Fusarium graminearum* F-1668	Isolated from rye forage (*Secale cereale*)	ARCM
*Fusarium oxysporum* f.sp. *radicis-lycopersici* ZUM2407 (*Forl* ZUM2407)	Tomato pathogen, the causal agent of tomato foot and root rot	Komissarov et al., 2026 [19]
*Fusarium oxysporum* f.sp. *radicis-cucumerinum* V03-2g (*Forc* V03-2g)	Tomato pathogen, the causal agent of cucumber foot and root rot	ARRIAM ** Komissarov et al., 2026 [19]
*Microdochium nivale* SPBSH	Isolated from infected winter wheat leaves	MGMM *** collection
*Trichoderma asperellum* F-1087	Biocontrol agent protecting plants via predation and parasitism mechanism	ARCM

* All-Russia Collection of Microorganisms, Puschino, Russia; ** ARRIAM All-Russia Research Institute for Agricultural Microbiology, Pushkin, Russia; *** The Laboratory of Molecular-Genetic and Microbiological Methods, Federal Research Center “Kazan Scientific Center of the Russian Academy of Sciences”, Kazan, Russia.

### 2.2. Rhizosphere Samples Collection and Isolation of Strains

Root and soil samples of *J. turcomanica* were collected from a mountainous region sierozem soil near the village of Makhtymkala in Turkmenistan (38.446173° N; 56.533288° E). Samples (1 g each) were suspended in 10 mL of PBS. Aliquots of 100 µL from serial dilutions 10 to 10^−5^ were spread-plated onto Gause’s No. 1 agar supplemented with nystatin. Plates were incubated at 28 °C for 14 days. Presumptive *Streptomyces* isolates were recovered by selecting colonies with characteristic actinomycete morphology (texture, pigmentation, and filamentous hyphae). These isolates were further purified to homogeneity.

### 2.3. Determination of Enzymatic Activity of the Isolated Strains

All activity tests were conducted in accordance with the previously described protocols, with minor adjustments [18]. Amylase activity was tested on Gause’s No. 1 agar. After incubation at 28 °C for 5 days, plates were poured with Lugol’s iodine solution (Biosintez, Penza, Russia). A clear zone of hydrolysis around colonies indicated amylase activity. Cellulase activity was assessed with MMA supplemented with 1% sodium carboxymethyl cellulose (CMC). After incubation at 28 °C for 5 days, plates were stained with 0.2% Congo Red for 15 min, followed by destaining with 1 M NaCl. Cellulase-positive isolates showed a zone of clearance around the colony. Protease activity was determined on MMA with addition of skim milk. A clear zone around colonies after 5 days of incubation at 28 °C indicated presence of protease activity. Lipase activity was determined by the opaque halo of calcium salts around colonies after incubation, indicating lipase production on a selective medium. Phytase activity was evaluated on phytate screening agar as a distinct clear zone around colonies. Hydrolytic activity was assessed qualitatively by the presence or absence of hydrolysis zones on selective media. No quantitative statistical analysis was applied to these data.

### 2.4. Ligninolytic Activity Analysis

The production of ligninolytic enzymes decolorizing methylene blue (MB) by selected *Streptomyces* isolates was estimated according to the method described earlier with some modifications [20]. Briefly 10 µL aliquots of seven-day-old cultures of each isolate were inoculated into 500 mL of medium—either fresh Gause’s No. 1 or King B, each supplemented with 20 µg/mL methylene blue. Flasks were incubated at 28 °C with agitation at 150 rpm for 14 days. After 7 and 14 days of incubation, samples were collected and centrifuged at 10,000 ×g for 10 min. Methylene blue degradation in the supernatants was determined spectrophotometrically by measuring the decrease in absorbance at 665 nm [21]. Non-inoculated media maintained under the same conditions served as the control. MB degradation (%) was calculated using the following formula:

$$Degradation\;rate\;\left(\%\right)=\frac{C-R}{C}\times 100$$

where “C” is the initial absorbance at 665 nm (uninoculated control), and “R” is the absorbance after incubation with the isolates. Degradation rates (%) were analyzed by two-way ANOVA with strain and medium/time as fixed factors, followed by Tukey’s multiple comparison test. This design allows comparison of isolates across two different media (King B and Gause’s No. 1) and two time points (7 and 14 days).

### 2.5. Assessment of Isolate Capability for Lignosulfonate Degradation

To assess the ability of the studied *Streptomyces* isolates to degrade lignosulfonates, they were incubated for 14 days at 28 °C in a lignosulfonate medium. After incubation, cells were removed by a two-step process: initial centrifugation at 13,000 ×g for 10 min, followed by filtration of the supernatant through 0.2 μm nitrocellulose filters (Millipore, Darmstadt, Germany). The particle size distribution of lignosulfonates in the final supernatants was measured using a BeNano 180 Zeta Pro spectrometer (Bettersize Instruments, Dandong, China). Measurements were performed at 21 °C and a scattering angle (θ) of 90° using monochromatic laser radiation (λ = 633 nm). The instrument’s measurement range was between 0.3 and200 nm. Autocorrelation functions were recorded, and data were processed using BeNano V3.0 software. As a control, a 0.5% (*w*/*v*) lignosulfonate solution, prepared in the same basal medium, was filtered through a nitrocellulose membrane prior to use [22].

### 2.6. Isolates Straw Degrading Activity Analysis

Lignin degradation in straw was quantified using the method as described [23]. Wheat straw (*Triticum aestivum*), collected in 2024 from standard agricultural wheat fields, was milled and used as the sole carbon source. A standard loop (10 μL) of a 7-day-old culture grown on Gause’s No. 1 agar was used to inoculate 100 mL of liquid minimal medium (MMB) supplemented with 100 mg of milled straw. Cultures were incubated in a shaking incubator at 120 rpm and 28 °C for 21 days. A control group, consisting of sterile medium with an equivalent amount of straw, was used to account for background abiotic degradation.

After incubation, the samples were dried at 80 °C. Subsequently, 5 mL of a concentrated nitric acid and 1,4-dioxane solution (1:4 *v*/*v*) was added to each sample. The samples were heated in a boiling water bath for 15 min, followed by the addition of 10 mL of 2 M NaOH. The mixture was then diluted to 50 mL with distilled water and filtered through filter paper. The absorbance of the filtrate was measured at 440 nm using a spectrophotometer (ALLSHENG, Hangzhou, China). Lignin content (absorbance at 440 nm) was analyzed by one-way ANOVA with treatment (strain) as the single fixed factor, followed by Tukey’s multiple comparison test.

### 2.7. Isolates Antagonistic Activity Analysis

Antagonistic activity was evaluated using a standard dual-culture plate assay. Briefly, mycelial plugs of the phytopathogenic fungi *Microdochium nivale*, *Fusarium oxysporum* f. sp. *lycopersici* (*Forl*), *Fusarium oxysporum* f. sp. *cucumerinum* (*Forc*), *Alternaria alternata*, and *Fusarium graminearum* were placed in the center of Petri dishes containing Gause’s No.1 agar medium. The *Streptomyces* isolates were inoculated as streaks or plugs at a distance of 4 cm from the pathogen. After incubation at 28 °C for 7 days, antagonistic activity was determined by measuring the growth inhibition zone of the pathogen facing the bacterial colony, indicating the production of antifungal metabolites.

The inhibition zones were measured from the edge of the pathogen colony to the edge of the bacterial colony using the Straight Line tool in ImageJ software 1.54g version (National Institutes of Health, Bethesda, MD, USA). Prior to measurement, images were calibrated using the known diameter of the Petri dish (90 mm) as a reference scale. All measurements were performed in three biological replicates, and data are presented as mean ± SD.

### 2.8. Isolates Identification by 16S rRNA Analysis

*Streptomyces* isolates were identified by sequencing a fragment of the 16S rRNA gene. Amplification of the full-length 16S rRNA gene fragment was performed with universal primers 27F (5′-AGAGTTTGATCCTGGCTCAG-3′) and 1522R (5′-AAGGAGGTGATCCAGCCGCA-3′) [24] using isolated chromosomal DNA as the matrix for amplification. The resulting amplicons were gel-purified using a Gel Extraction Kit (Thermo Scientific, Waltham, MA, USA). The nucleotide sequence of the purified DNA fragment was determined using Sanger sequencing (Evrogen, Moscow, Russia). The resulting sequences were assembled using the Clone Manager 9.0 software (Westminster, CO, USA) and analyzed using the NCBI BLAST web interface (https://blast.ncbi.nlm.nih.gov, accessed on 15 August 2025). The isolates were identified based on the highest sequence similarity to strains of known species from the GenBank database.

A phylogenetic tree was constructed in MEGA 11 software. The 16S rRNA gene sequences obtained in this study were aligned with the closest type strains. Sequences were retrieved from the NCBI GenBank database using the ClustalW algorithm implemented in MEGA 11. Phylogenetic relationships were inferred using the neighbor-joining (NJ) method based on the Kimura 2-parameter model. The reliability of the tree topology was assessed by 1000 bootstrap replicates. *Rhodococcus erythropolis* DCL14 (AJ131637.1) was used as the outgroup.

### 2.9. Statistical Analysis

All experiments were performed with three biological replicates per sample, except where otherwise stated. Data are presented as mean ± standard deviation (SD). Statistical analyses were performed using GraphPad Prism version 9.0 (GraphPad Software, San Diego, CA, USA) or an equivalent software package. The significance threshold was set at *p* < 0.05 for all tests. In the figures, differences that did not reach the significance threshold (*p* < 0.05) are marked as “NS” (non-significant).

## 3. Results

### 3.1. Isolation and Morphological Characterization of Streptomyces from the Juniperus turcomanica Rhizosphere

Using a selective isolation medium for actinobacteria, multiple bacterial isolates were obtained from the rhizosphere of *J. turcomanica* near the village of Makhtymkala in Turkmenistan. From these, seven isolates were selected based on morphological traits characteristic of the genus *Streptomyces* (filamentous hyphae, spore formation, and typical colony texture), as well as the stability and reproducibility of these traits after repeated subculturing. These isolates were designated as ATM9, ATM14, ATM15, ATM16, ATM18, ATM19, and ATM24 (Table 2).

**Figure 1:**
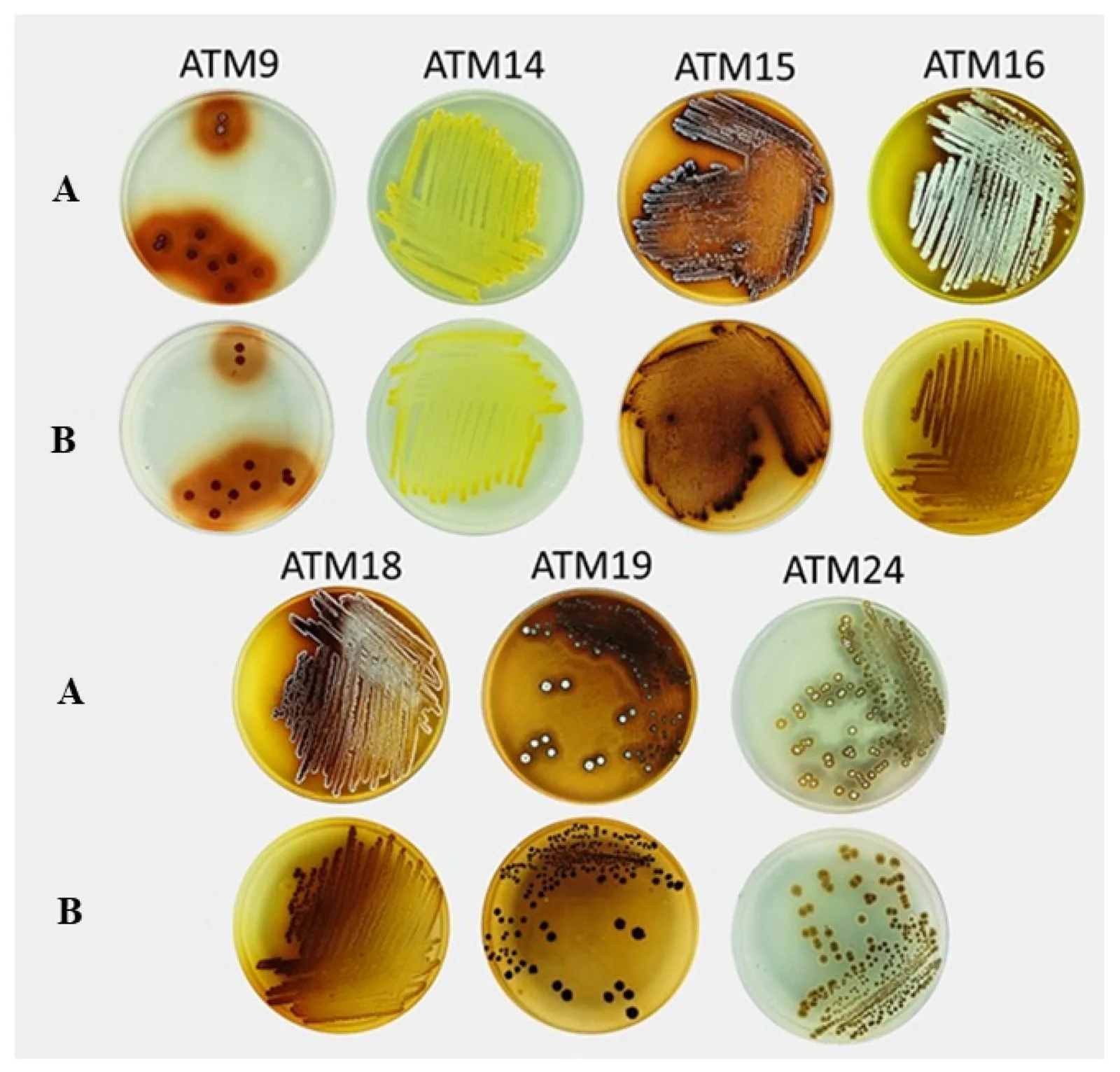
Morphology of *Streptomyces* colonies on Gause’s No. 1 agar after 7 days of incubation at 28 °C. (**A**) Top view of Petri dishes. (**B**) Bottom view of Petri dishes.

Pigment production is a notable feature, with five isolates (ATM9, ATM15, ATM16, ATM18, ATM19) producing diffusible pigments of various colors (red-brown, yellow-brown, light brown, dark brown) (Figure 1).

Pigments produced by *Streptomyces* often have diverse biological functions. These include antimicrobial activity, which may be associated with the ability to inhibit the growth of competing microorganisms. Melanin-like pigments are thought to contribute to the organism’s stress tolerance and protection against UV radiation. Notably, isolates ATM14 and ATM24 were distinguished by the absence of diffusible pigment production during growth on the isolation medium.

### 3.2. Enzymatic Activity Screening of the Isolated Strains

For further isolate characterization, we employed plate assay methods using specialized media to check the enzymatic potential of the seven selected *Streptomyces* isolates (ATM9, ATM14, ATM15, ATM16, ATM18, ATM19, and ATM24). Each class of enzyme production was detected by the formation of clear or colored zones around the substrate mycelium, indicating substrate hydrolysis (Table 3).

All seven isolates exhibited cellulolytic and lipolytic activity (Table 3). Amylase activity was detected in all isolates except ATM14 and ATM19. Proteolytic activity was present in all isolates except ATM24. Phytase activity was detected in all isolates except ATM9. Among the isolates, ATM15, ATM16, and ATM18 possessed all five hydrolytic activities tested, whereas ATM9, ATM14, ATM19, and ATM24 were positive for four of the five activities (Table 3). Overall, the seven isolates displayed a broad spectrum of hydrolytic activities, with each isolate showing a distinct enzymatic profile.

### 3.3. Methylene Blue Decolorization by the Isolate Strains

**Figure 2:**
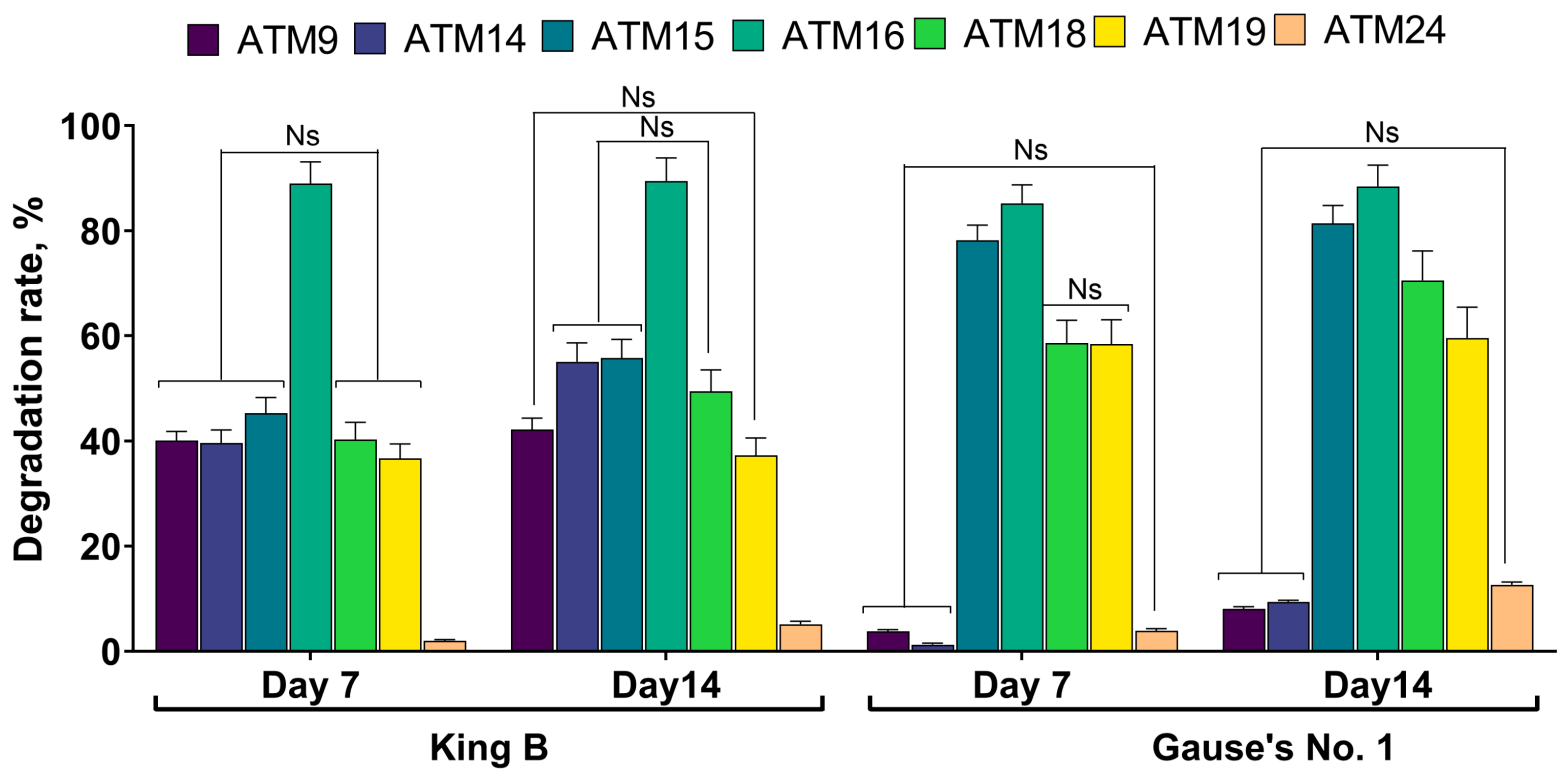
Degradation of methylene blue by *Streptomyces* isolates after 7 and 14 days of incubation at 28 °C on King’s B and Gause’s No. 1 media. Data are expressed as the mean ± standard deviation (SD). To assess the statistical significance of differences, two-way ANOVA followed by Tukey’s multiple comparisons (*p* < 0.05, *n* = 3). “NS” (nonsignificant) showed differences below the significance threshold of *p* < 0.05.

Methylene blue (MB) decolorization is a widely used assay for assessing ligninolytic potential under different substrate conditions. All isolates except ATM24 showed the ability to decolorize MB, suggesting the presence of ligninolytic activity (Figure 2).

On the nutrient-rich King B medium, the isolate ATM16 demonstrated the highest activity in decolorization, reaching 89% after 7 days and 89.6% after 14 days of incubation, indicating rapid and efficient degradation of the dye within the first week. However, in the nutrient-poor Gause’s No. 1 medium, a different pattern emerged. Isolates ATM9 and ATM14 showed reduced decolorization compared to their performance on King B medium. In contrast, isolates ATM15, ATM18, and ATM19 exhibited slightly higher activity in Gause’s No. 1, with increased decolorization ranging from 18% to 35% compared to their performance on the richer medium. Notably, the isolate ATM16 maintained consistently high activity in Gause’s No. 1, achieving 84.9% decolorization after 7 days and 88.1% after 14 days. These values were comparable to those observed on King B medium, and the highest among all isolates tested for both media.

### 3.4. Estimation of Isolates Strains for Lignosulfonate Utilization

**Figure 3:**
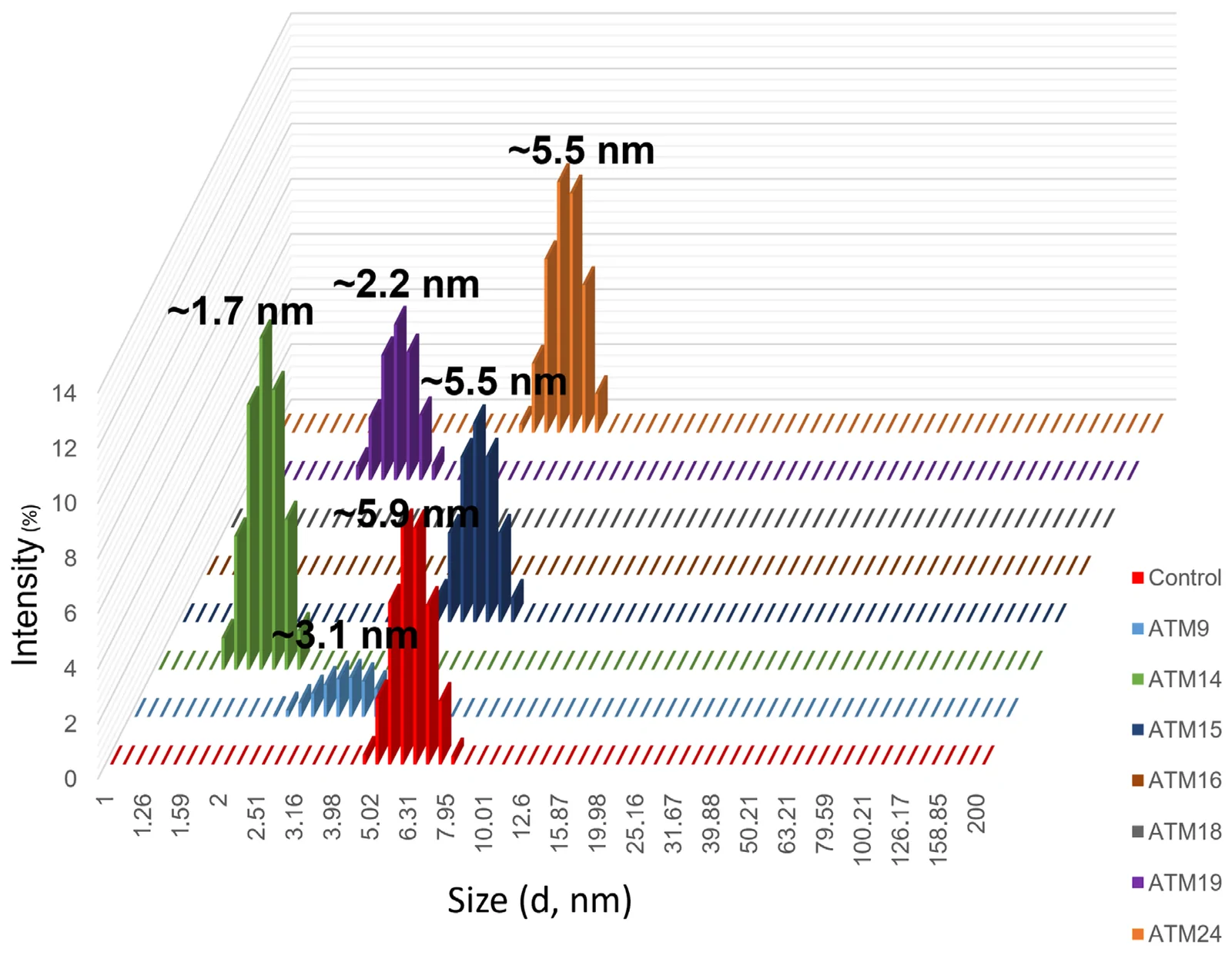
Degradation of lignosulfonate assessed by dynamic light scattering (DLS) in supernatants of the bacterial strains after 14-day incubation.

Lignosulfonates are the primary byproducts of sulfite pulping that consist of water-soluble sulfonated lignin derivatives. To assess the ligninolytic potential of the isolated strains, we analyzed lignosulfonate particle sizes following incubation with each isolate. In the control samples lignosulfonate particle size was approximately 5.9 nm. Isolates ATM15 and ATM24 showed no substantial degradation, leaving residual particle sizes of 5.5 nm and 5.3 nm, respectively (Figure 3).

These values were comparable to the data obtained for the control group. Moreover, isolates such as ATM9, ATM14, and ATM19 demonstrated significant degradation, reducing particle sizes to 3.1 nm, 1.7 nm, and 2.2 nm, respectively. At the same time, the most pronounced degradation was observed with isolates ATM16 and ATM18. Lignosulfonate particles were below the detection limit of the instrument, suggesting substantial degradation of the substrate and confirmation by chemical analysis is required.

### 3.5. Evaluation of the Straw Degradation by Isolate Strains

**Figure 4:**
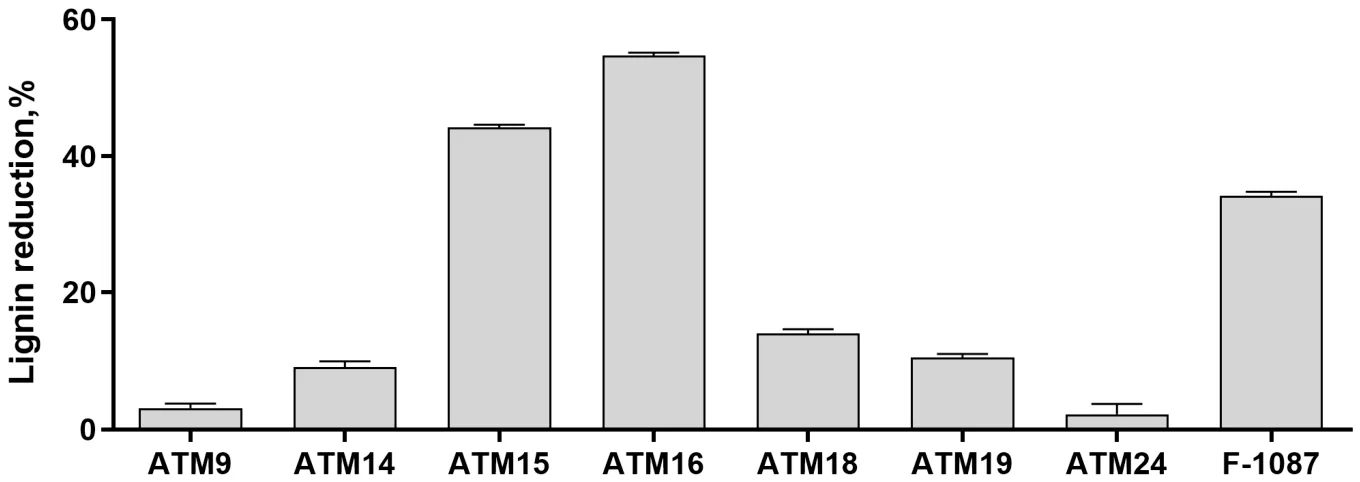
Lignin degradation in straw by *Streptomyces* isolates. Ctrl—Without microbial inoculation (negative control); F-1087—*Trichoderma asperellum* (positive control). Data are expressed as the mean ± standard deviation (SD). To assess the statistical significance of differences, one-way ANOVA followed by Tukey’s multiple comparisons (*p* < 0.05, *n* = 3).

Developing a reliable and standardized method for quantifying lignin degradation in complex substrates, such as straw, that was exposed to various physicochemical and biological degradation factors was essential for our comparative study. We optimized a previously described spectrophotometric method for lignin quantification and standardized its extraction protocol from the straw that had been harvested from the standard agricultural wheat field. This approach minimized variability and enabled a targeted comparison of lignin degradation among our seven *Streptomyces* isolates (ATM9–ATM24). Following incubation under uniform conditions, several isolates induced small but statistically significant reductions in lignin content relative to controls. Specifically, isolates ATM9, ATM14, ATM18, ATM19 and ATM24 decreased lignin concentrations by 3.1%, 9.1%, 14%, 10.5% and 2.2% respectively, compared to the control. However, isolates ATM15 and ATM16 exhibited substantially higher lignin-degrading activity, reducing its content by 44.2% and 54.8%, respectively, compared to the negative control. Moreover, these two isolates also showed significantly higher lignin-degrading activity in comparison to the positive control (*Trichoderma asperellum*) (Figure 4).

Our findings suggest that the ATM15 and ATM16 isolates have unique enzymatic capabilities that allow for more efficient lignin breakdown. These isolates, when combined, show promising activity in lignin degradation. Further investigation is needed to validate this potential and to determine if these isolates could be useful tools for lignin degradation.

### 3.6. Determination of Antagonistic Activity of the Isolate Strains

**Figure 5:**
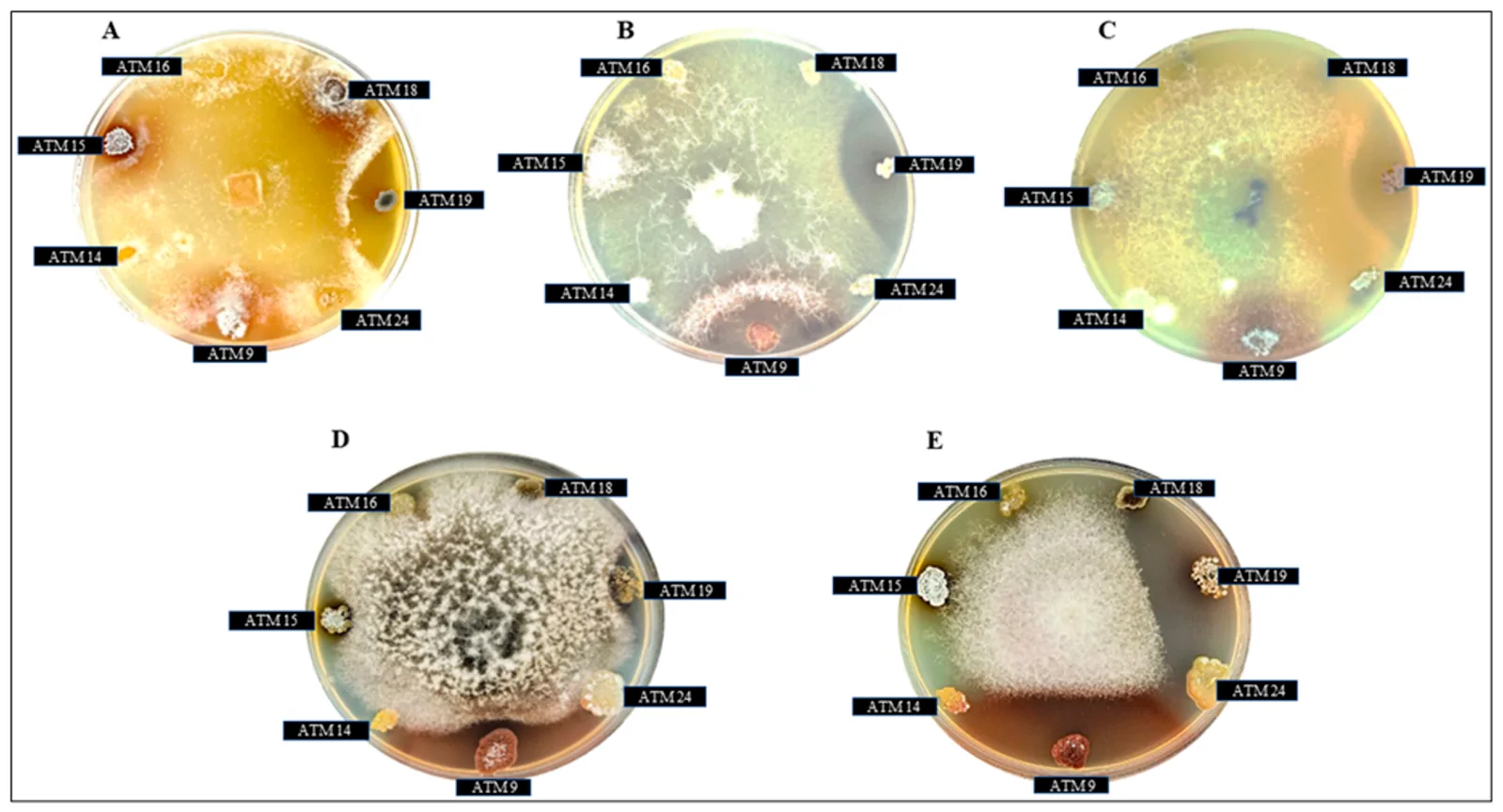
Antifungal activity of *Streptomyces* isolates against phytopathogenic fungi. (**A**) *M. nivale*. (**B**) *Forl*. (**C**) *Forc*. (**D**) *A. alternata.* (**E**) *F. graminearum*.

The ability of plant-associated microorganisms to suppress phytopathogens (antagonistic activity) is likely essential for plant protection, especially in symbionts of long-lived species like *J. turcomanica.* To evaluate this, we assessed the antifungal activity of *Streptomyces* isolates by measuring the inhibition zones they produce against a panel of five phytopathogenic fungi (Table 4, Figure 5).

Isolate ATM19 exhibited the broadest antifungal spectrum, inhibiting all five pathogens with inhibition zones ranging from 1.0 ± 0.2 mm (*A. alternata*) to 12.4 ± 0.2 mm (*F. graminearum*). Isolate ATM9 inhibited four of the five fungi, with the largest zone against *F. graminearum* (9.8 ± 0.3 mm), followed by *Forl* (6.4 ± 0.2 mm), *A. alternata* (3.2 ± 0.4 mm), and *M. nivale* (1.3 ± 0.2 mm). Isolate ATM15 showed activity against two fungi, with inhibition zones of 1.3 ± 0.1 mm (*M. nivale*) and 1.2 ± 0.2 mm (*A. alternata*). Isolate ATM24 inhibited only *F. graminearum* (4.3 ± 0.2 mm), while isolate ATM14 showed marginal activity against *F. graminearum* (1.4 ± 0.2 mm). Isolates ATM16 and ATM18 did not exhibit detectable antagonistic activity under the tested conditions.

### 3.7. Taxonomic Identification of Isolates

Taxonomic identification of the isolates, based on comparative analysis of 16S rRNA gene sequences, revealed that the microbial isolates belonged to the genus *Streptomyces*. The results of taxonomic analysis are presented in Table 5.

**Figure 6:**
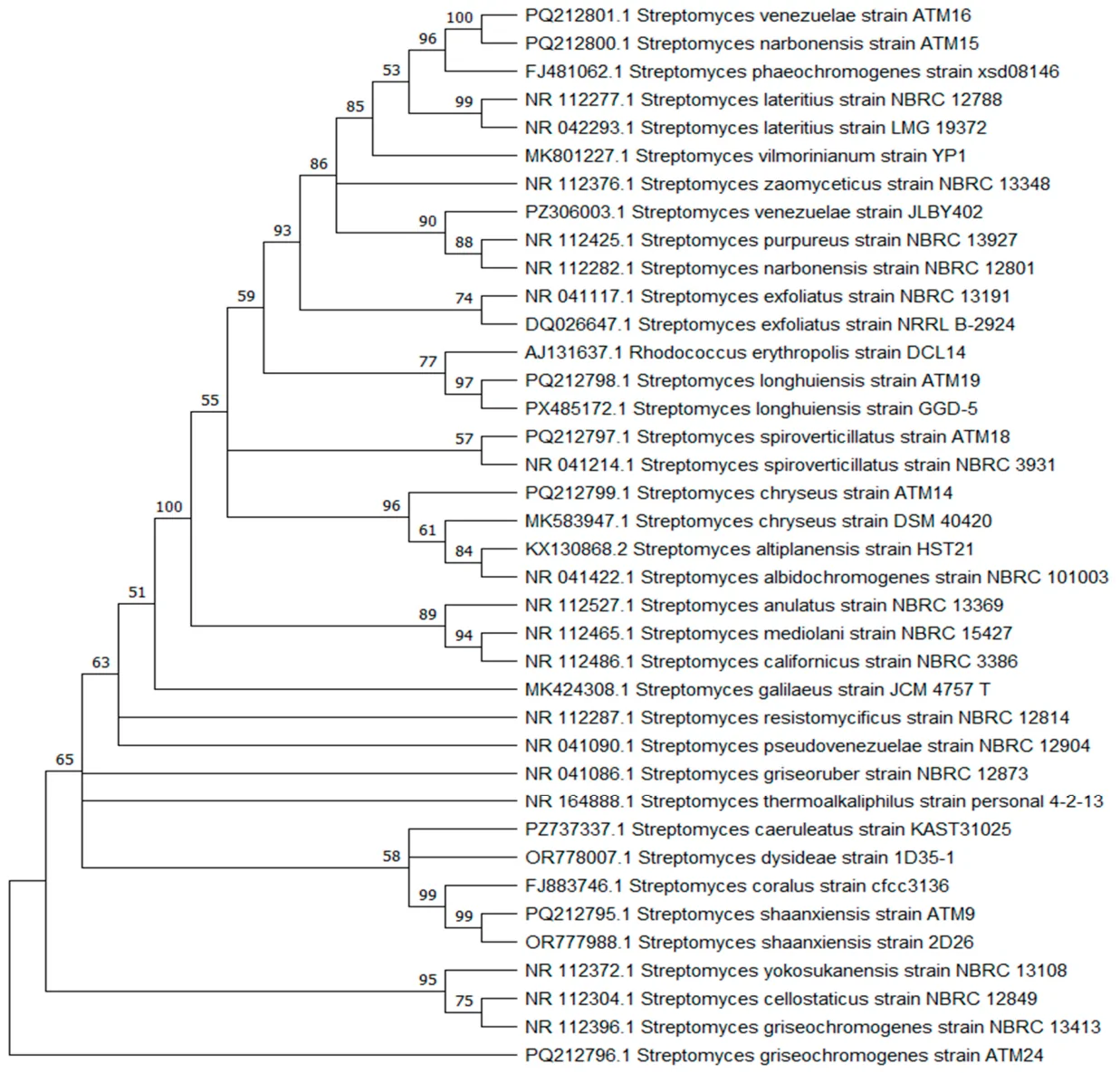
Phylogenetic tree based on 16S rRNA gene sequences showing the relationships among seven isolates (ATM9, ATM14, ATM15, ATM16, ATM18, ATM19, ATM24) and their closest *Streptomyces* relatives. *Rhodococcus erythropolis* DCL14 (AJ131637.1) was used as the outgroup. Bootstrap values (>50%, 1000 replicates) are shown at branch nodes. Scale bar: substitutions per site.

The highest similarity values were observed for isolates ATM14 and ATM15, which show 99.72% similarity to *S. chryseus* DSM 40420 (MK583947.1) and *S. narbonensis* NBRC 12801 (NR_112282.1), respectively. Isolate ATM16 shows 99.66% similarity to *S. venezuelae* ATCC 15068 (CP029194.1). Isolates ATM18, ATM9, and ATM19 display 99.37%, 99.36%, and 99.23% similarity to *S. spiroverticillatus* NBRC 3931 (NR_041214.1), *S. shaanxiensis* CCNWHQ 0031 (NR_116681.1), and *S. longhuiensis* BH-MK-02 (CP085173.1), respectively. In contrast, isolate ATM24 shows only 97.43% similarity to its closest type strain, *S. griseochromogenes* ATCC 14511(T) (MN686613.1). This value is below the generally accepted threshold for species delineation within the *Streptomyces* genus, which is typically considered to be ≥99%. This result suggests that ATM24 may represent a potentially novel species, but additional evidence is needed to confirm this status. The 16S rRNA gene sequences generated in this study have been deposited in GenBank with accession numbers PQ212795–PQ212801 (Table 5). The phylogenetic analysis revealed that the isolates are distributed across several distinct clades, generally consistent with the results of the similarity search. Isolates ATM15 and ATM16 formed a monophyletic group with 100% bootstrap support, which clustered together with *S. narbonensis* NBRC 12801 and *S. venezuelae* JLBY402, also with 100% support (Figure 6). These findings suggest that ATM15 and ATM16 may be closely related and represent the same species.

Isolate ATM9 clustered with *S. shaanxiensis* 2D26 with 99% bootstrap support, forming a well-supported clade that also included *S. coralus* cfcc3136 and *S. dysideae* 1D35-1 (Figure 6). Similarly, isolate ATM19 grouped with *S. longhuiensis* GGD-5 with 97% bootstrap support, and isolate ATM14 clustered with *S. chryseus* DSM 40420 with 96% bootstrap support. These results are consistent with the high 16S rRNA similarity values (≥99.2%) obtained for these isolates. In contrast, the phylogenetic position of isolate ATM18 was less robust. Although it clustered with *S. spiroverticillatus* NBRC 3931, the bootstrap support for this node was only 57% (Figure 6). This indicates that the placement of ATM18 is not well resolved, and its precise taxonomic affiliation remains uncertain based on 16S rRNA data alone. Finally, isolate ATM24 occupied an isolated position in the tree, with an anomalously long branch (0.48881) that was approximately two orders of magnitude greater than the branch lengths of other taxa (Figure 6). This finding, combined with its low similarity in 16S rRNA (97.43%), supports the hypothesis that ATM24 may represent a distinct and potentially novel species within the *Streptomyces* genus. However, confirmation of this status requires further data, such as whole-genome sequencing with ANI/dDDH calculations or multilocus sequence analysis (MLSA).

### 3.8. Functional Complementarity of the Isolates

The *Streptomyces* isolates from the *J. turcomanica* rhizosphere exhibited complementary functional traits (Table 6). Although the isolates were tested individually and no co-culture experiments were performed, their combined capabilities suggest potential functional complementarity, encompassing substantial lignin degradation (ATM16, ATM18), efficient breakdown of native lignocellulose (ATM15, ATM16), broad-spectrum antifungal defense (ATM19, ATM9), and specialized enzymatic functions including lipase (ATM14, ATM18) and phytase (ATM16, ATM24).

The consortium of the mentioned isolates may play a vital role in the health and longevity of its host plant by providing both nutrient cycling and pathogen protection functions. The isolates, particularly ATM16 and ATM15 (both exhibiting all five hydrolytic activities and efficient at lignin degradation) and ATM19 (exhibiting broad-spectrum antifungal activity), hold significant promise for biotechnological applications in bioremediation, biofuel production, and biocontrol. Moreover, isolate ATM24, a potentially novel species with 97.43% similarity to *S. griseochromogenes*, warrants further genomic characterization and may represent an undiscovered taxon with unique properties.

## 4. Discussion

Plant protection is an essential task throughout all stages of plant development, including senescence and subsequent decomposition. Without proper management, crop residues serve as a source of nutrients for phytopathogenic microorganisms, allowing them to survive and persist until a new host is available. Biodegradation of crop residues in the absence of effective microbial antagonists can actually increase the level of phytopathogens in soils, which then negatively affects subsequent crop yields. The effective degradation of plant residues presents unique challenges, as these substrates are exposed to more intense and fluctuating abiotic stresses, including desiccation, extreme temperatures, and UV radiation. Therefore, microorganisms that are able to not only survive but also maintain metabolic activity under such harsh conditions are required in order to ensure efficient decomposition of residues and nutrient cycling, ultimately contributing to the enhancement of soil fertility. Consequently, identifying and utilizing beneficial soil microorganisms that provide both properties and suppress phytopathogens is a promising strategy for reducing pathogens and promoting a healthy soil microbiome [25]. For this study, an ecological niche was selected in the rhizosphere of *J. turcomanica* (Turkmen juniper), a long-lived plant native to the arid mountainous regions of Turkmenistan. Soil samples from this area are subject to extreme temperature fluctuations, including significant heating and cooling, which act as strong selective pressures on associated microorganisms. We propose that these mutualistic interactions are based on bacterial processing and colonization of senescent plant tissues from the host. In turn, the bacteria contribute to the host’s mineral nutrition and provide biocontrol against phytopathogens. It was hypothesized that such an environment would select for stress-tolerant and beneficial microorganisms with high biotechnological and agricultural potential. All microorganisms isolated in this study from the rhizosphere of *J. turcomanica* were identified as members of the genus *Streptomyces* (Table 3). These strains are non-pathogenic and represent promising candidates for use as biological control agents in environmental systems. In addition, *Streptomyces* is a well-known genus with multiple beneficial traits, including phytostimulation [26], bioprotection [27] and the production of extracellular hydrolytic enzymes that degrade cell wall components of phytopathogenic microorganisms [28]. Furthermore, some *Streptomyces* strains produce lignin-degrading enzymes, cellulases, and amylases that are essential for utilizing senescent or dead plant tissues. Additionally, actinomycetes can survive in extreme conditions such as desiccation and high temperatures due to their ability to form resilient spores [29]. Positive correlations between pigmentation and antimicrobial activity were observed. Pigmented strains such as ATM9, ATM15, and ATM19 tended to have higher antimicrobial activity than non-pigmented strains (ATM14, ATM24). However, there were some exceptions: pigmented strains ATM16 and ATM18 showed lower antifungal activity. These results indicate that the biosynthesis of pigments may be interconnected or regulated together with the production of antimicrobial secondary metabolites in a variety of strains. However, this relationship is not consistent across all isolates. In addition to the core activities characteristic of *Streptomyces*, this study has identified several strain-specific functional traits. Strains ATM16 and ATM24 exhibit phytase activity, which is not widely found among *Streptomyces*. Phytase activity helps to hydrolyze phytate and increase the bioavailability of phosphorus in soil, making it more accessible for plant uptake [30]. Furthermore, strains ATM9, ATM15, ATM16, and ATM18 demonstrate a broad spectrum of enzymatic activities, including amylase, protease, cellulase, and phytase production. These enzymes contribute to soil fertility by breaking down organic matter and increasing biological availability. The combined action of the produced enzymes establishes the strains as valuable contributors to soil fertility through the decomposition of organic matter and mobilization of nutrients. Among these strains, *S. narbonensis* is closely related to our ATM15 and is known for producing the macrolide antibiotic narbomycin. Narbomycin inhibits the growth of Gram-positive bacteria and has potential applications in medicine [31]. According to our data, the isolated strain ATM15 showed inhibition of *M. nivale* and *A. alternata* and might be used as a biocontrol agent against phytopathogenic fungi for crop protection. Strains ATM9 and ATM19, identified as *S. shaanxiensis* and *S. longhuiensis* respectively, represent strong candidates for use both as plant growth stimulants and as biocontrol agents. This study provides the first report of its enzymatic and antifungal activity against a number of phytopathogens: *M. nivale*, *Forl*, *Forc*, *A. alternata*, and *F. graminearum*. Newly introduced species *S. shaanxiensis* has remained without detailed functional or metabolic characterization [32,33]. Along with antifungal properties, strain *S. longhuiensis* (ATM19) exhibited significant lignin-degrading activity, including the degradation of both native lignin and its derivatives. The combination of lignocellulolytic and antifungal traits makes strain ATM19 a strong candidate for various applications, ranging from residue decomposition to phytopathogen suppression. In addition, a quantitative methylene blue decolorization assay confirmed the synthesis and activity of ligninolytic enzymes in isolates ATM9, ATM14, ATM15, ATM16, ATM18, and ATM19. This demonstrates their ability to degrade lignin and its derivatives. The findings are consistent with the dynamic light scattering (DLS) data obtained for sodium lignosulfonate degradation. Among the tested isolates, ATM16, ATM18, and ATM19 showed the highest ligninolytic activity.

Furthermore, a high ligninolytic activity of strain ATM16 is consistent with previous observations. Ehrlich et al. firstly demonstrated that *S. venezuelae* can grow in the presence of low concentrations of phenol, which typically appears in the process of lignin biodegradation [34]. This might support the findings regarding high ligninolytic potential of ATM16. Similarly, *S. spiroverticillatus*, a species closely related to ATM18, has been shown to decolorize the polymeric dye Poly R-478 and degrade rice and wheat straw, recovering acid-precipitated lignin [35]. Our results underscore the efficiency of lignin degradation by the representatives of *S. spiroverticillatus* as well. The functional complementarity observed among the isolates suggests their potential role as a functionally complementary set of strains in the *J. turcomanica* rhizosphere, although synergistic interactions remain to be tested experimentally. Isolate ATM16 exhibits high activity across multiple enzyme classes and a remarkable ligninolytic capacity, as demonstrated by substantial degradation of lignosulfonates (with particle sizes below the DLS detection limit) and a 54.8% reduction in native straw lignin content. The strain ATM15 demonstrated exceptional capacity in breaking down native lignocellulose, reducing lignin by 44.2%, while also exhibiting moderate antagonistic activity against plant pathogens. ATM18 demonstrated significant potential for lignin degradation, as evidenced by substantial degradation of lignosulfonate (particles below the DLS detection limit). We hypothesize that strains ATM19 and ATM9 may perform a protective function by exhibiting broad-spectrum antifungal activity against plant pathogens. Notably, strain ATM24 showed promise as a phosphorus mobilization specialist with high phytase activity. Furthermore, ithe 16S rRNA gene sequence similarity between strain ATM24 and its closest type strain is onlyt.43% s,itouggests inghat it may represent a novel taxon that warrants further investigation.

## 5. Conclusions

This study demonstrates that the rhizosphere of *J. turcomanica* harbors diverse microorganisms with potential for application in agriculture and biotechnology. The seven selected strains (ATM9, ATM14, ATM15, ATM16, ATM18, ATM19, and ATM24) exhibited diverse enzymatic activities and antimicrobial properties, suggesting their potential use as a functionally complementary synthetic consortium. However, synergistic interactions remain to be tested. The combined lignocellulolytic, antifungal, and plant growth-promoting properties of these isolates make them promising candidates for integrated pest management and sustainable agriculture. Their ability to decompose plant residues while simultaneously suppressing phytopathogens addresses several challenges in crop production, such as nutrient recycling and disease control. Further research should focus on optimizing the composition of synthetic consortia based on these strains, validating their efficacy under field conditions, and elucidating the molecular mechanisms underlying their interactions.

## Figures and Tables

**Table 2:** Morphological features of ATM isolates.

Isolates	Colony Morphology	Colony Margin	Substrate Mycelium Color	Aerial Mycelium Color	Diffusible Pigment
ATM9	Round	Entire	Burgundy	Bright gray	Red-brown
ATM14	Round	Scalloped	Yellow	White	None
ATM15	Concentric	Entire	Black	Whitish	Yellow-brown
ATM16	Round	Scalloped	Brown	Cream	Light brown
ATM18	Round	Scalloped	Brown to black	White	Dark brown
ATM19	Round	Scalloped	Nearly black	Cream	Light brown
ATM24	Round	Entire	Light brown	White	None

**Table 3:** Qualitative hydrolytic activity of *Streptomyces* isolates.

Isolates	Cellulase	Amylase	Protease	Lipase	Phytase
ATM9	+	+	+	+	-
ATM14	+	-	+	+	+
ATM15	+	+	+	+	+
ATM16	+	+	+	+	+
ATM18	+	+	+	+	+
ATM19	+	-	+	+	+
ATM24	+	+	-	+	+

Legend: “+”—activity detected; “-”—no activity detected.

**Table 4:** Inhibition zones (radius, mm) of *Streptomyces* isolates against phytopathogenic fungi in dual-culture plate assays.

Isolates	*M. nivale*	*Forl*	*Forc*	*A. alternata*	*F. graminearum*
ATM9	3.2 ± 0.4	6.4 ± 0.2	-	3.2 ± 0.4	9.8 ± 0.3
ATM14	-	-	-	-	1.4 ± 0.2
ATM15	1.3 ± 0.1	-	-	1.2 ± 0.2	-
ATM16	-	-	-	-	-
ATM18	-	-	-	-	-
ATM19	7.6 ± 0.2	8.9 ± 0.1	8.5 ± 0.2	1.0 ± 0.2	12.4 ± 0.2
ATM24	-	-	-	-	4.3 ± 0.2

Legend: Values are inhibition zones (radius, mm) presented as mean ± SD (*n* = 3). “-” indicates no detectable activity.

**Table 5:** Taxonomic identification of bacterial isolates based on 16S rRNA gene sequence analysis.

Isolate Name	Similarity Percentage	Strain with Maximal Identity in the NCBI Database	GenBank Accession Numbers of 16S rRNA Gene Sequences
ATM9	99.36%	*Streptomyces shaanxiensis* strain CCNWHQ 0031 (NR_116681.1)	PQ212795
ATM14	99.72%	*Streptomyces chryseus* strain DSM 40420 (MK583947.1)	PQ212799
ATM15	99.72%	*Streptomyces narbonensis* strain NBRC 12801 (NR_112282.1)	PQ212800
ATM16	99.66%	*Streptomyces venezuelae* strain ATCC 15068 (CP029194.1)	PQ212801
ATM18	99.37%	*Streptomyces spiroverticillatus* strain NBRC 3931 (NR_041214.1)	PQ212797
ATM19	99.23%	*Streptomyces longhuiensis* strain BH-MK-02 (CP085173.1)	PQ212798
ATM24	97.43%	*Streptomyces griseochromogenes* strain ATCC 14511(T) (MN686613.1)	PQ212796

**Table 6:** Ranking of strains by key functions.

Rank	Lignin Degradation	Antifungal Activity	Enzymatic Versatility	Overall Biotechnological Potential
**1**	ATM16 (54.8% straw; substantial LS degradation)	ATM19 (5/5 pathogens)	ATM16 (5/5 enzymes)	ATM16 (lignin + enzyme powerhouse)
**2**	ATM15 (44.2% straw)	ATM9 (4/5 pathogens)	ATM15 (5/5 enzymes)	ATM15 (lignin + moderate defense)
**3**	ATM18 (substantial LS degradation; 14% straw)	ATM15 (2/5 pathogens)	ATM18 (5/5 enzymes)	ATM19 (defense specialist)
**4**	ATM19 (10.5% straw)	ATM24 (1/5 pathogen)	ATM24 (4/5 enzymes)	ATM18 (lipase + LS degradation)
**5**	ATM14 (9.1% straw)	ATM14 (1/5 pathogen, marginal)	ATM9, 14, 19 (4/5 enzymes)	ATM9 (defense + moderate enzymes)
**6**	ATM9 (3.1% straw)	ATM16, 18 (none)	—	ATM14 (lipase specialist)
**7**	ATM24 (2.2% straw)	—	—	ATM24 (phytase + potentially novel taxon)

## Data Availability

The data supporting the findings of this study are available within the article. The 16S rRNA gene sequences reported here have been deposited in the NCBI GenBank database under accession numbers PQ212795–PQ212801. Further inquiries can be directed to the corresponding author.
